# Investigating the Role of Osteopontin (OPN) in the Progression of Breast, Prostate, Renal and Skin Cancers

**DOI:** 10.3390/biomedicines13010173

**Published:** 2025-01-13

**Authors:** Gautam Kundu, Selvakumar Elangovan

**Affiliations:** School of Biotechnology, Kalinga Institute of Industrial Technology (KIIT) Deemed to be University, Bhubaneswar 751024, Odisha, India; gkundu84@gmail.com

**Keywords:** breast cancer, prostate cancer, renal cancer, skin cancer, osteopontin, transcriptomics, splice variants, oncogenes, pathway analysis

## Abstract

**Background/Objectives**: Cancer is caused by disruptions in the homeostatic state of normal cells, which results in dysregulation of the cell cycle, and uncontrolled growth and proliferation in affected cells to form tumors. Successful development of tumorous cells proceeds through the activation of pathways promoting cell development and functionality, as well as the suppression of immune signaling pathways; thereby providing these cells with proliferative advantages, which subsequently metastasize into surrounding tissues. These effects are primarily caused by the upregulation of oncogenes, of which SPP1 (secreted phosphoprotein 1), a non-collagenous bone matrix protein, is one of the most well-known. **Methods**: In this study, we conducted a further examination of the transcriptomic expression profile of SPP1 (Osteopontin) during the progression of cancer in four human tissues, breast, prostate, renal and skin, in order to understand the circumstances conducive to its activation and dysregulation, the biological pathways and other mechanisms involved as well as differences in its splicing patterns influencing its expression and functionality. **Results**: A significant overexpression of SPP1, as well as a set of other highly correlated genes, was seen in most of these tissues, indicating their extensive implication in cancer. Increased expression was observed with higher tumor stages, especially in renal and skin cancer, while applying therapeutic modalities targeting these genes dampened this effect in breast, prostate and skin cancer. Pathway analyses showed gene signatures related to cell growth and development enriched in tumorigenic conditions and earlier cancer stages, while later stages of cancer showed pathways associated with weakened immune response, in all cancers studied. Moreover, the utilization of therapeutic methods showed the activation of immunogenic pathways in breast, prostate and skin cancer, thereby confirming their viability. Further analyses of differential transcript expression levels in these oncogenes showed their exonic regions to be selectively overexpressed similarly in tumorigenic samples in all cancers studied, while also displaying significant differences in exon selectivity between constituent transcripts, providing a basis for their high degree of multifunctionality in cancer. **Conclusions**: Overall, this study corroborates the entrenched role of SPP1 in the progression of these four types of cancer, as confirmed by its overexpression and activation of related oncogenes, their co-involvement in key cellular pathways, and predisposition to exhibit differential splicing between their transcripts, while the above effects were found to be highly inhibitable through treatment methods, thereby highlighting its promising role in therapeutic development.

## 1. Introduction

Cancer is one of the most globally prevalent diseases currently, affecting millions of individuals worldwide. It is primarily characterized by the abnormal and uncontrolled growth and division of cells, which results in the creation of aggregates of cells known as tumors, in affected tissues. Tumorous cells are capable of proliferating irrepressibly and subsequently migrating into neighboring tissue to affect healthy cells, and inducing similar actions in them in a condition known as malignancy; following which, fully developed tumors invade other tissues via circulatory and lymphatic systems, causing metastasis [1]. The principal mechanism causing initial tumor formation is the disruption of equilibrium in the expression between two classes of genes: proto-oncogenes, which are positive regulators of DNA replication and cell division, and tumor-suppressor genes which negatively regulate these processes in cells containing any defects or putative tumor-affected cells, thereby preventing them from undergoing division. However, the occurrence of external carcinogenic factors such as chromosomal translocations, genetic mutations and amplifications or epigenetic changes [2] can facilitate the activation and transformation of proto-oncogenes into oncogenes that display abnormally higher expression. This is typically accompanied by the downregulation of tumor-suppressor genes, leading to the loss of homeostatic balance in affected cells and commencing in their unrestrained cell division and resultant tumor formation and growth facilitated by angiogenesis, while inhibiting normal cell apoptosis, thus promoting the growth and proliferation of tumors. Fully metastasized tumors, which represent later stages of cancer and during which symptoms are most apparent, are highly resistant to regular treatment methods. This causes cancer to have a very low survival rate. Additionally, as a result, it is found to be more prevalent in countries with a low socio-economic development status, due to a lack of access to adequate diagnostic and treatment facilities for larger sections of society as compared to developed nations.

One of the most well-known oncogenes in cancer is SPP1 (secreted phosphoprotein 1, which encodes the protein Osteopontin (OPN), a non-collagenous bone matrix protein belonging to the Small Integrin-Binding Ligand N-linked Glycoprotein (SIBLING) family of glycoproteins, which plays an extensive role in several stages of tumor development [3], wherein its increased secretion has been found to be correlated with higher risks of malignancy in various forms of cancer [4]. It binds to the plasma membrane, primarily through its signaling receptor CD44 and the integrin receptor αvβ3 and has a diverse range of cellular functionalities. SPP1 is involved in the development of the conducive tumor microenvironment (TME) during initial tumor formation through angiogenesis and upregulation of various cellular processes including cell proliferation, adhesion, migration and inter-cellular communication [5,6]. Epithelial–mesenchymal transition (EMT) has been found to be mediated by SPP1, especially in its type 3 form (cancer metastases), through the upregulation of transcription factors such as TWIST [7,8], SNAIL1 [8] and SNAIL2 [8], as well as the activation of pathways such as PI3K-AKT-TWIST and hypoxia-inducible factor-1 alpha (HIF-1α) in various cancer models. It has been found to promote endothelial cell selection and migration during tumor angiogenesis [9,10]. Another of its mechanisms of action in cancer includes the negative regulation of T-cell activation through the overexpression of inhibitory molecules such as PD-L1, which results in T-cell exhaustion and apoptosis, causing immunosuppression in the tumor microenvironment and promoting tumor growth [11,12,13]. Finally, it has also been found to play an important role in the development of tumor resistance to treatment by chemotherapy and radiotherapy through the regulation of several pathways such as autophagy, PI3K/Akt signaling, MAPK signaling and EGFR pathways [14]. All these properties of SPP1 make it an interesting target with a large range of purposes, including as a useful biomarker for diagnosis of tumor aggressiveness and disease stage and survival in affected individuals [4,15,16]; as well as the development of therapeutic approaches involving SPP1 inhibition to suppress tumor growth, proliferation, and metastasis in patients [17,18,19,20].

The perturbation of proto-oncogenes and their conversion into oncogenes, as well as their subsequent role in cancer progression, is typically governed by the activity of a variety of biological pathways involving them, which principally enable their functionality. The positive regulation of several tumorigenic pathways associated with cell cycle progression, cell proliferation and cell apoptosis is brought about through the overexpression and genetic alterations occurring in specific ‘driver’ genes, with many of them being co-occurrent in multiple forms of cancer [21]. Among these, SPP1 is a notable one, and is found to be similarly involved in a large repertoire of tumorigenic pathways, during which it is highly correlated with a set of specific other oncogenes in tumorous cells [8]. Gaining further insight into their pathway enrichment profiles is therefore crucial in order to investigate the precise mechanisms of oncogenic activity in disease progression within human tissues.

Additionally, the functionality of genes is also determined by the expression profiles of their constituent ‘transcripts’, or isoforms created by splicing patterns. Due to the high variability of the RNA splicing process, there exists the possibility of a gene having multiple splice variants or protein isoforms, occurring due to selective inclusions and deletions in exonic regions within each isoform, causing them to vary in sequence composition and length after undergoing translation to proteins. As a result, there can be a differential expression between spliced isoforms for the same gene, which ultimately influences their functionality as well as factors such as stability, localization within the genome and liability to transcriptional regulation [22]. Oncogenic activity in cancer has also been found to be associated with abnormalities in the regulation of alternative splicing, forming larger numbers of differentially expressed transcripts and thereby driving higher protein diversity and functionality in cancer progression [23], as well as the development of resistance to treatment modalities such as chemotherapy and radiotherapy [24]. A primary factor driving these variations in splicing events is differential exon usage (DEU) occurring during the splicing process, which is observed in 90–95% of all genes with multiple transcripts [25]. Exonic regions may show differences in usage between the transcripts of an oncogene. The analysis of these DEU patterns, which may occur during tumorigenesis, can potentially provide greater insights into their functions and roles in cancer progression.

In this study, we have conducted a detailed investigation of the role of SPP1 in four particular types of cancer in humans, breast, prostate, renal and skin, which not only have a higher global prevalence but also wherein the role and effects of SPP1 have already been extensively studied [13,19,26,27]. We have specifically focused on studying patterns of overexpression for SPP1 and some highly related oncogenes, their pathway enrichment profiles as well as their effects in influencing differential splicing of transcripts, occurring during tumor formation and development, or in response to application of therapeutic modalities, in these four cancers. We aim to build further upon pre-existing knowledge about this oncogene and delve further into its association with cancer by analyzing its expression profile in these tissues during tumor occurrence and progression, its effects on the enrichment of related cellular pathways as well as examining differences in the expression of its splicing variants as a potential cause of modifications in its functionality during tumorigenesis.

## 2. Materials and Methods

### 2.1. Experimental Design

**Figure 1:**
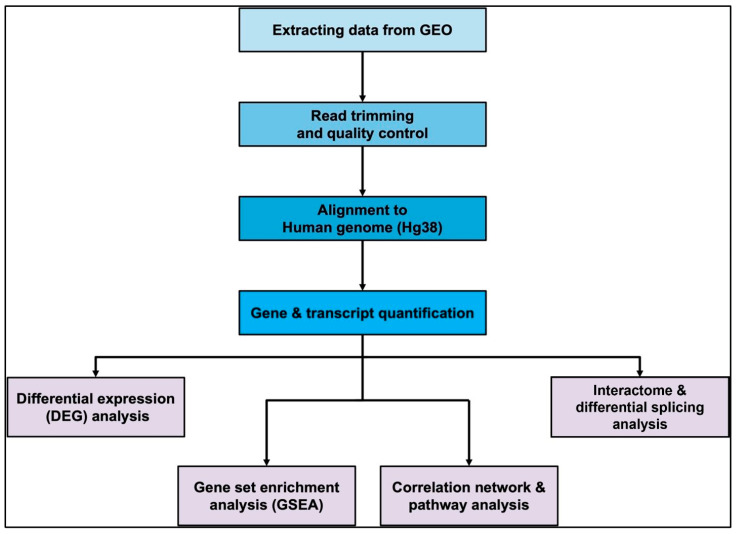
Pipeline for transcriptomic data analyses.

Bulk transcriptomic (paired-end total RNA-Seq) data were collected from the Gene Expression Omnibus (GEO) repositories of 17 previous cancer studies [28,29,30,31,32,33,34,35,36,37,38,39,40,41,42,43,44,45] in the aforementioned four types of tissues. These datasets were appropriately selected from studies involving a sufficient number of experimental treatment and control samples. Sequenced data were obtained in the form of sequence read archive (SRA) files for a total of 390 samples (all samples were sourced directly from the GEO repositories of their study of origin, which also include information regarding status of acquired written consent. References to the same are specified in Supplementary Tables S1–S4), which were then processed using a specifically designed transcriptomic analysis pipeline (Figure 1).

### 2.2. Data Collection and Pre-Processing

SRA files for all samples were converted into the FASTQ format using the NCBI tool ‘fasterq-dump’ (v2.9.1) [46], which was followed by trimming reads using Trimmomatic (v0.39) [47] in order to remove adapter sequences and filter out reads having a Phred quality score (Q) below 30 and lengths shorter than 50 base pairs. Quality scores were additionally verified using FASTQC (v0.11.8) [48], which showed all samples to have reads with good quality (Q > 30). Subsequently, reads were aligned using Hisat2 (v2.1.0) [49] to the human genome (Hg38 Ensembl v97) in paired-end unstranded mode, following which uniquely mapped reads, in BAM format, were quantified for human gene and transcript features using the above reference genome, using Subread featureCounts (v1.6.4) [50]. Read counts per feature were aggregated for all samples per dataset to create matrices, to be subsequently used in primary analyses using R.

### 2.3. Primary Analyses and Techniques

The read count matrices obtained were normalized using the TMM method and subjected to differential gene expression (DEG) using the R package ‘edgeR’ (v3.36.0) [51]. Genes with uniformly low expression across samples were removed using the default filtering method in edgeR (count ≥ 10 in at least 15 samples). Specific blacklisted genes such as intronic genes, pseudogenes and non-coding RNA, whose expression might correspond to background noise arising due to biological variation, were also excluded from count matrices. Samples were classified into groups based on conditions in their study of origin, representing tumor occurrence, grade, disease stage, treatment modalities or other physiological factors. Using the glmQLFit function from the edgeR package, count matrices were fit onto generalized linear models and comparisons between categorized sample groups were performed using QLF (quasi-likelihood F) tests, obtaining genes showing dysregulation in either direction. Highly significant genes were selected using cutoff values of absolute FC > 1.5 along with nominal *p*-value < 0.05.

Gene set enrichment analyses (GSEA v4.0) [52] were conducted with the normalized count matrices for determining pathways (represented by gene signatures) showing enrichment in each sample group; using the c2 (canonical pathways) and c6 (oncogenic signatures) gene set families obtained from the MSigDB database [53]. Since SPP1 has been found to interact with specific other oncogenes during cancer progression, we also used a set of six highly correlated genes found in a breast cancer study (FAP, ACTA2, TWIST1, ITGB1, S100A4 and CXCL12) [8], and looked for enrichment of MSigDB gene sets which involved SPP1 and at least one among these interacting genes. Significantly enriched pathways were determined using cutoff values of absolute NES (normalized enrichment score) > 1.4 along with a nominal *p*-value < 0.05. Lists of the top 100 significantly dysregulated genes obtained in each DEG comparison per study, which showed patterns of co-expression with SPP1, were selected for analysis using Metascape (v3.5.20240101) [54] in order to identify the top 20 functionally enriched pathways by their Gene Ontology (GO) terms, as well as the interactions between them listed in order of significance.

Differential splicing analyses were performed using the R package ‘DEXSEQ’ (v1.40.0) [55]. Aligned reads in BAM format were first quantified for exonic regions within Ensembl transcripts per gene in the reference genome (Hg38 Ensembl v97), and the resultant ‘DEXSEQ’ count matrices were then subjected to comparisons using a negative binomial likelihood ratio test (LRT) between the categorized DEG groups as before, to examine differences in selective utilization of regions between the constituent transcripts per gene. Regions with significant differential exon usage (DEU) were identified using the default threshold of a Benjamini–Hochberg (FDR) corrected *p*-value of less than 0.05.

## 3. Results

A total of 390 samples were processed successfully using the described pipeline (Figure 1) for four types of cancer-affected human tissue (Table 1). Quality control metrics displayed an average of 90% reads retained after filtering for low-quality bases, out of which around 75% reads were uniquely mapped to the Hg38 genome. Assignment of reads showed more than 70% mapped reads matching genomic features. Normalized and filtered count matrices obtained were grouped according to sample metadata for breast cancer (Table S1), prostate cancer (Table S2), renal cancer (Table S3) and skin cancer (Table S4) for downstream comparisons. After the final qualitative filtering for undesirable genes and those with uniformly low expression, about 75–80% genes were retained in the count matrix.

**Figure 2:**
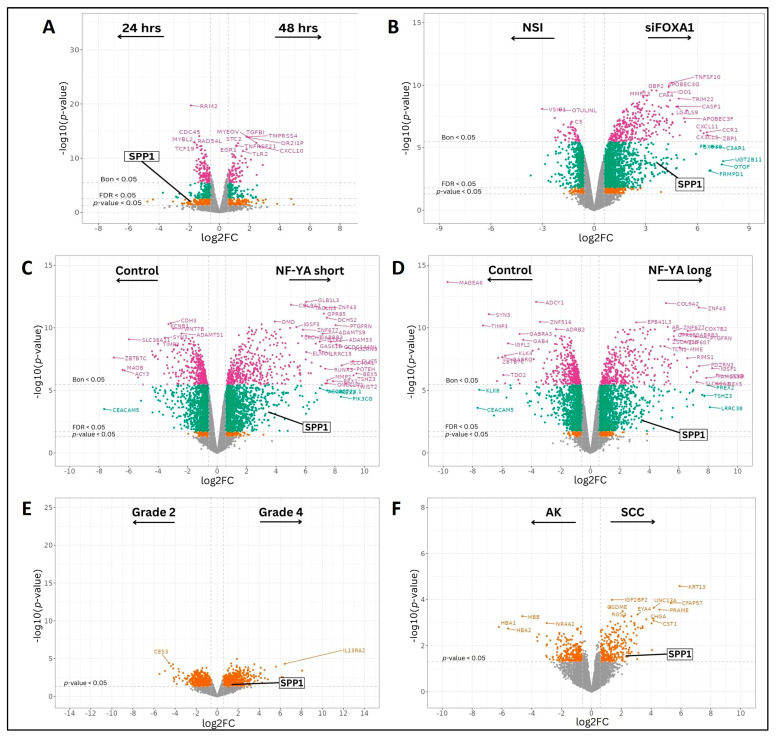
Significant genes using differential gene expression (DEG) analyses in (**A**) breast cancer; (**B**–**D**) prostate cancer; (**E**) renal cancer; and (**F**) skin cancer datasets. Colors indicate significance categories: Genes showing no significant change in expression (gray), genes with nominal *p*-value < 0.05 (orange), Benjamini–Hochberg (FDR) corrected *p*-value < 0.05 (green), Bonferroni corrected *p*-value < 0.05 (purple). Dashed lines indicate cutoff values for significance: Absolute log2FC > 0.585 (corresponding to FC > 1.5), and nominal *p*-value < 0.05.

In the breast cancer study GSE213474 [28], MCF7 cells treated with IFN-γ stimulation showed SPP1 to be significantly downregulated in samples (log2FC = −2.067, *p*-value = 0.038), when the treatment was applied for a longer duration (48 h v/s 24 h) (Figure 2A). When analyzing PC-3 cell samples in the prostate cancer study GSE193127 [36] which were transfected with siRNAs targeting FOXA1, a known prostate oncogene, SPP1 was expressed significantly higher (log2FC = 4.005, *p*-value = 1.658 × 10^−4^), as compared to control samples which were transfected with non-targeting siRNA (Figure 2B). Prostate PC-3 cells in the study GSE179990 [37] showing an overexpression for nuclear transcription factor NF-YA, in both its long and short alternatively spliced isoforms also displayed higher SPP1 levels, with the shorter isoform ‘NF-YAs’ or ‘NFYAv2’, of length 318 bp, having greater significance (log2FC = 3.673, *p*-value = 6.501 × 10^−4^) (Figure 2C) than the longer isoform ‘NF-YAl’ or ‘NFYAv1’, of length 347 bp (log2FC = 3.578, *p*-value = 7.989 × 10^−4^) (Figure 2D).

When comparing renal cell carcinoma (RCC) tissue samples from the dataset GSE151419 [40] with increasing Fuhrman grades, there was a significantly large increase observed in SPP1 expression between grade 4 and grade 2 cancer-affected samples (log2FC = 1.590, *p*-value = 0.046) (Figure 2E). Finally, upregulation was showed in a skin cancer dataset (GSE84293) [44] within samples affected by cutaneous squamous cell carcinoma (cuSCC) (log2FC = 2.603, *p*-value = 0.022), as compared to samples affected by actinic keratosis (AK) (Figure 2F), which is a benign condition characterized by skin lesions, and may act as a precursor to cancer.

**Figure 3:**
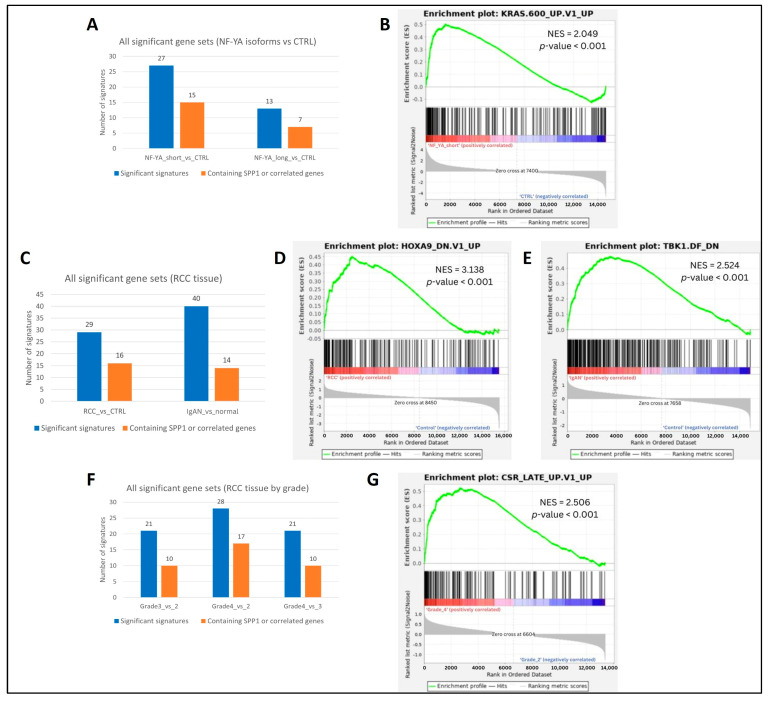
Results from GSEA analyses: (**A**): significantly enriched signatures in a prostate cancer dataset; (**B**) top signature in NFYA (short isoform) expressing cells v/s controls; (**C**) signatures in two renal cancer datasets; (**D**,**E**) top signatures in RCC cells v/s controls, and IgA nephropathy v/s normal tissue, respectively; (**F**) significantly enriched signatures with increasing tumor grades in RCC tissue; (**G**) top signature in Grade 4 RCC v/s Grade 2. Colors in bar plots indicate the total number of signatures (blue) and number of signatures containing SPP1 and at least one of its six top interacting genes (orange). Enrichment plots from GSEA show the enrichment score (ES) obtained per gene in the signature, with the location of the peak ES value indicating the direction of overall enrichment for the signature. Red and blue colors indicate positive and negative enrichment respectively.

Our GSEA analyses confirmed patterns of significant enrichment for significant pathways (gene signatures) in samples showing high dysregulation in SPP1 and other oncogenes, concordantly as found in DEG analyses. PC-3 cells in the prostate cancer dataset GSE179990 [37] showed greater overall enrichment in samples expressing the shorter isoform (318 bp long) of NF-YA, than its longer isoform (347 bp long) (Figure 3A), with the top signature being ‘KRAS.600_UP.V1_UP’ (NES = 2.049, *p*-value < 0.001) (Figure 3B). In renal cancer, tumorous samples from RCC tissue in the dataset GSE167573 [38] enriched a larger number of signatures than controls (Figure 3C), with the highest being ‘HOXA9_DN.V1_UP’ (NES = 3.138, *p*-value < 0.001) (Figure 3D), while samples affected by IgA nephropathy in GSE141295 [39] also showed more enrichment than unaffected controls (Figure 3C), with the top signature being ‘TBK1.DF_DN’ (NES = 2.524, *p*-value < 0.001) (Figure 3E). In another renal cancer dataset, GSE151419 [40], samples showed a general surge in the number of enriched signatures with higher tumor grades, from grade 2 to grade 4 (Figure 3F). The signature ‘CSR_LATE_UP.V1_UP’ was highly enriched at grade 4 (NES = 2.506, *p*-value < 0.001) (Figure 3G).

**Figure 4:**
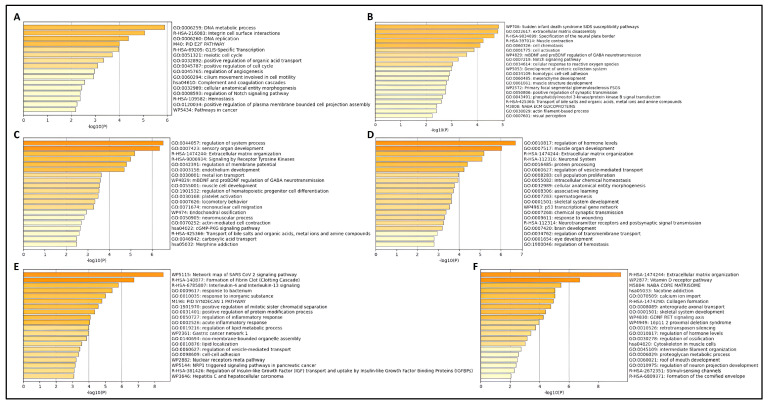
Results from Metascape analyses showing pathways involving significant DEGs, in (**A**): breast cancer; (**B**,**C**): prostate cancer; (**D**,**E**): renal cancer; and (**F**): skin cancer datasets. The top 20 pathways are shown in descending order of significance, ranked by *p*-value, with darker-colored bars indicating pathways with greater significance.

Metascape analyses using top significant DEGs expressed unidirectionally with SPP1, and its interacting partner genes also showed similar results in pathway involvement. In the breast cancer dataset GSE213474 [28], many pathways related to cell growth and division were found to involve genes that had shown a significant downregulation with the application of IFN-γ treatment (Figure 4A), including integrin cell surface interactions, a significant hallmark of SPP1 action. Prostate cancer samples in GSE179990 [37], with high expression for NFYA (both its long and short isoforms), showed similar pathways to be present within highly upregulated genes (Figure 4B and Figure 4C, respectively). In renal cancer, tumorous samples in GSE167573 [38] displayed a large number of cellular developmental pathways (Figure 4D), including extracellular matrix organization, another key feature of SPP1 functionality. Samples in GSE151419 [39] affected by renal tumors of higher grades also had similar effects, with several pathways within genes higher in grade 4 RCC being related to the regulation of cellular processes (Figure 4E). Finally, similar effects were seen in the skin cancer dataset GSE113113 [42,43], within genes expressed higher in SCC samples (Figure 4F) as compared to samples with actinic keratosis (AK).

**Figure 5:**
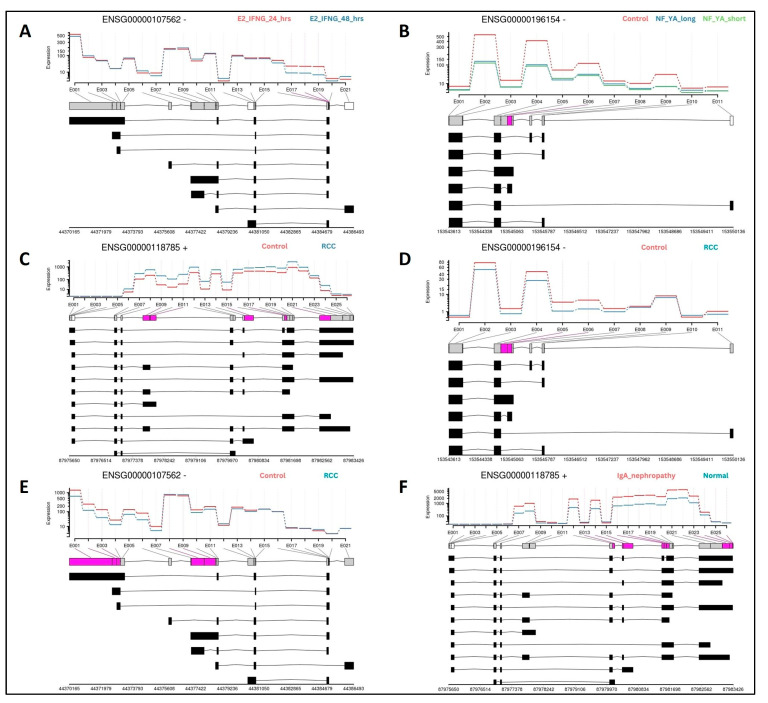
Results from DEU analyses showing exonic regions present selectively across transcripts for (**A**): CXCL12 in breast cancer; (**B**) S100A4 in prostate cancer; (**C**) SPP1; (**D**) S100A4 and (**E**) CXCL12, in renal cancer (RCC); and (**F**) SPP1 in another renal cancer dataset (IgA nephropathy). Graphs show expression levels in exonic regions for each pair of conditions (colored red and blue) per gene, and lines beneath denote the primary gene and its constituent transcripts (spliced isoforms). Boxes within each line indicate exonic regions per transcript, and are colored by significance categories: exons with non-significant DEU (gray), exons absent in the primary gene but present in any transcript and with significant DEU (white) and those with presence in the primary gene and significant DEU (pink).

In differential exon usage (DEU) analyses, significant changes in selectivity were observed for SPP1 and many of its interacting partner genes, in conditions consistent with DEG and pathway analysis results. In the breast cancer study GSE213474 [28], the second set of MCF7 cell samples, which were supplemented with heat-inactivated FBS (HD) along with estradiol (E2), showed three exonic regions (E017, E018 and E019) of CXCL12 or ‘ENSG00000107562’, to differ significantly with longer time under treatment using IFN-γ stimulation (Figure 5A). In the prostate cancer dataset GSE179990 [37], PC-3 cells overexpressing NF-YA (both its short and long isoforms) showed one exon (E006) to be changed across transcripts of S100A4, or ‘ENSG00000196514’ (Figure 5B). Cells from renal cell carcinoma (RCC) tissue in the dataset GSE167573 [38] showed tumorous samples to have multiple exonic regions in several genes, among them SPP1 or ‘ENSG00000118785’ with five exons (Figure 5C), S100A4 with two exons (Figure 5D) and CXCL12 with five exons (Figure 5E). SPP1 was also observed to have 10 exons significantly different in IgA nephropathy-affected samples from RCC tissue in the dataset GSE141295 [39] (Figure 5F) compared to normal cortex samples.

## 4. Discussion

The activation of proto-oncogenes by external carcinogenic factors and their resultant conversion into tumor-causing oncogenes has been well-observed to be the root cause of the development and subsequent progression of cancer in various human tissues. It can be brought about by their overexpression in tumorous cells, as well as other effects such as the presence of mutations or genetic alterations, or fusions occurring with other genes. They also have the ability to influence normal cellular pathways and capture them, utilizing their activity to facilitate critical cellular functions which promote their selective initial growth and development in tumorous cells, provide themselves with a proliferative advantage, maintain a supportive tumor environment and ultimately favor tumor invasion and metastasis to other cells and tissues [56]. Here, we sought to further investigate the multidimensional role of one of the most prominent oncogenes, SPP1, that encodes the protein Osteopontin in human tissues, including its functional mechanisms during cancer progression, its pathway enrichment landscape as well as structural and other changes occurring in this gene and some of its highly co-expressed genes interacting with it that drive the same processes. Finally, we also wanted to determine these changes occurring in response to particular therapeutic modalities of cancer treatment. As evidenced by a greater abundance of pre-existing literature on the effects of SPP1 in tumor progression as well as a potential therapeutic target [13,19,26,27], combined with a generally higher global prevalence of cancer in four particular human tissues, breast, prostate, renal and skin, we specifically focused on studying the above effects in these types of cancer.

### 4.1. Breast Cancer

The application of treatment using IFN-γ stimulation was able to produce a considerable suppression in SPP1. This effect was further enhanced with treatment duration, with samples on long-term treatment showing significantly reduced expression of SPP1 and many of its highly correlated oncogenes. GSEA analyses also confirmed this outcome, with the number of significantly enriched gene signatures reducing with a longer time spent on treatment, indicating rapid suppression in tumor activity, an effect also seen using Metascape analyses, with pathways related to cell growth and division being downregulated with time on treatment. In DEU analyses, the largest effect in exonic region differences across transcripts, with treatment duration was seen in CXCL12, an oncogene closely related to SPP1.

### 4.2. Prostate Cancer

Samples showed a high synergistic association in expression patterns between SPP1 and the oncogene NF-YA, wherein samples that overexpressed this gene also had increased SPP1 levels. The shorter isoform of NF-YA also showed greater enrichment in gene signatures than the longer isoform, which potentially indicates its greater susceptibility to carcinogenic activation and thereby more significant involvement in eventual cancer progression. DEU effects were also observed most strongly in these samples. Transfection with another oncogene FOXA1 was also found to cause significant upregulation in SPP1 expression. In both cases, there was an upregulation of cellular pathways concerning cell growth, differentiation and extracellular matrix organization; all of them are hallmarks of tumor progression. On the contrary, applying treatment using knockdown of the oncogenes BRCA1 and TP53 was able to bring significant downregulation in SPP1.

### 4.3. Renal Cancer

In this type of cancer, samples showed considerable changes in SPP1 expression with higher disease stage, as represented by Fuhrman grades, with the number of gene signatures increasing as well. Higher stages were observed to influence the activation of pathways implicated in reduced serum response, which potentially indicated a weakening of the immune system in cases of late-stage cancer, wherein tumor cells have already undergone metastasis. The occurrence of external conditions conducive to tumorigenesis, such as IgA nephropathy, displayed a simultaneous involvement of other oncogenes such as KRAS, which contributed towards the upregulation of several other host genes, as indicated by common pathways. Similar to prostate cancer, tumor-affected samples also showed several cellular developmental pathways to be upregulated. DEU patterns were much more noticeable as compared to breast and prostate cancer, with SPP1 as well as its interacting partners S100A4 and CXCL12 showing significant differences in exon usage in tumor-affected samples.

### 4.4. Skin Cancer

Similar to renal cancer, samples in skin cancer also showed significant overexpression of SPP1 with higher stages. Application of treatment using the antibody CPI-670242, an inhibitor of the oncogene LSD1 was found to induce SPP1 downregulation. Similar pathway enrichment profiles were observed as in the other three types of cancer, with pathways pertaining to cell growth and differentiation being upregulated with tumorigenic conditions and suppressed with the action of targeted therapeutic modalities involving oncogenic inhibition.

### 4.5. Summary

In general, all four types of cancer studied showed an activation of SPP1 as well as some of its highly correlated oncogenes; that is, its interacting partners co-involved in tumor progression. Several cellular pathways, many of which included the above oncogenes, showed strong association with tumorigenesis in conjunction with patterns of overexpression. On the other hand, the application of treatment modalities was able to induce a number of immune response pathways, implying a restoration of the natural immune system, thereby validating immunotherapy to be a promising method of cancer treatment.

Analyses of differential isoform expression in oncogenes showed changes in exon selectivity within their constituent ‘transcripts’ or spliced isoforms, to occur concurrently with their patterns of dysregulation and functional enrichment, as well as indicating them as significant drivers of these effects. Variations in exonic region usage between transcripts of several oncogenes were seen to correspond well with changes in their actual expression levels. Since even minor changes in exonic expression have been observed to correlate with considerable differences in overall gene expression and resultant functionality, it also highlights the potential use of therapeutic methods such as exon and gene therapy to target these effects and induce selective splicing within specific oncogenes and editing their transcriptional ability in cells.

Overall, through our study, we have been able to supplement previous results by confirming the effects and patterns of SPP1 overexpression during tumorigenic conditions [6] as well as its suppression in response to external therapeutic modalities [13], at various stages of the disease, using an adequate number of samples from multiple bulk RNA-seq studies. By investigating the pathway enrichment landscape of this gene, in addition to some other known oncogenes which have been previously found to be highly correlated with its expression in human cancer tissues, in the above conditions, we propose our results to serve as a validation of the functional role that SPP1 plays in the four types of cancer studied. Through our supplementary analyses of differential splicing patterns of SPP1 and its interacting oncogenes, we add an additional layer of detail to previous findings by confirming the variations in functionality that occur in conjunction with oncogenic overexpression in conditions conducive to cancer progression or in response to treatment methods imparted, thereby further validating SPP1 to be a biomarker as well as a potential therapeutic target in the types of cancer studied [17,18].

### 4.6. Conclusions

In conclusion, the results of our analyses confirmed the multifaceted role of SPP1 in several aspects of cancer progression, thus establishing it as one of the most potent oncogenes. It also displayed considerable responses to various treatment methods, thereby potentially opening new avenues for its diagnostic utilization as a biomarker and in the further development of therapeutic modalities such as immunotherapy and targeted therapy, as well as novel and upcoming methods such as gene therapy.

### 4.7. Limitations and Future Perspectives

This research, however, is subject to several limitations. One of the primary shortcomings of this study was the lower sample size for certain studies in some of the cancer types studied. In part due to the lower availability of high-throughput total RNA-seq samples from GEO datasets, the statistical robustness of our analyses was constrained by having a smaller number of samples. In our DEG analyses, since we were aiming to obtain a maximum number of differentially expressed genes to be used in further downstream analyses, we had set our significance threshold values at typically low levels. Moreover, the methods used for pathway analyses in GSEA (v4.0) and Metascape (v3.5.20240101) were also based on online databases, which undergo constant updating and refinement, because of which possibilities exist for obtaining better outcomes with these tools in the future. For our differential splicing analyses, the R package ‘DEXSEQ’ was one of the only tools available for bulk RNA-seq data, which calculates significance using likelihood ratio tests with a fitted GLM model and uses a higher significance threshold of *p*-value < 0.05. The use of newer or improved tools to perform this analysis could show clearer results of DEU patterns. In addition, the utilization of single-cell transcriptomics data, which have greater specificity to individual tissue types, could also potentially show more biologically relevant as well as statistically significant results.

There has also been a significant interest in the association between SPP1 and reduced immunity in several diseases including cancer in humans. Enhanced levels of Osteopontin (OPN) secretion in the bone marrow have been observed to initiate immune responses such as emergency granulopoiesis during tumorigenic conditions, which eventually lead to disruption in the equilibrium between lymphoid and myeloid cells, causing immunosuppression and inhibition of apoptosis [57]. However, the exact mechanisms of these effects are not known yet. High OPN levels have also been linked to increased pathogenesis in several autoimmune disorders such as Allergic Contact Dermatitis (ACD) [58], which may also be exacerbated by potential multiple variants of SPP1 that arise due to differential splicing effects [59]. Therefore, there exist additional possibilities for the development of Osteopontin as a disease biomarker, as well as a therapeutic target for additional diseases besides cancer as well.

## Figures and Tables

**Table 1:** Bulk RNA-seq samples used in analyses per tissue type.

Tissue	Number of Studies	Samples Obtained
Breast	5	78
Prostate	5	101
Renal	4	96
Skin	3	115
Total	17	390

## Data Availability

The original contributions presented in the study are included in the article/Supplementary Materials, further inquiries can be directed to the corresponding author.

## Supplementary Materials

The following supporting information can be downloaded at: https://www.mdpi.com/article/10.3390/biomedicines13010173/s1, Table S1: Samples analyzed for breast cancer; Table S2: Samples analyzed for prostate cancer; Table S3: Samples analyzed for renal cancer; Table S4: Samples analyzed for skin cancer.

## List of Abbreviations

αVβ3	Alpha-v Beta-3
ACTA2	Actin alpha 2
AK	Actinic keratosis
AKT	AKR thymoma protein kinase
BAM	Binary alignment map
BRCA1	Breast cancer type 1
CD44	Cluster of differentiation 44 molecule
cuSCC	Cutaneous squamous cell carcinoma
CXCL12	C-X-C Motif Chemokine Ligand 12
DEG	Differential gene expression/Differentially expressed genes
DEU	Differential exon usage
DEXSEQ	Differential exon usage analysis in RNA-seq (R package)
DNA	Deoxyribonucleic acid
EGFR	Epidermal growth factor receptor
EMT	Epithelial mesenchymal transition
ES	Enrichment score
FAP	Fibroblast activation protein
FASTQ	Fast adaptive shrinkage threshold algorithm with quality score
FBS	Fetal bovine serum
FC	Fold change
FDR	False discovery rate
FOXA1	Forkhead box A1
GEO	Gene expression omnibus
GLM	Generalized linear model
GO	Gene Ontology
GSEA	Gene set enrichment analysis
HIF	Hypoxia-inducible factor
IFN-γ	Interferon gamma
ITGB1	Integrin subunit beta
KRAS	Kirsten rat sarcoma virus
LRT	Likelihood ratio test
MAPK	Mitogen-activated protein kinases
NCBI	National Center for Biotechnology Information
NES	Normalized enrichment score
NF-YA	Nuclear transcription factor Y subunit alpha
OPN	Osteopontin
PD-L1	Programmed death-ligand 1
PI3K	Phosphoinositide 3-kinase
QLF	Quasi-likelihood f test
RCC	Renal cell carcinoma
RNA	Ribonucleic acid
S100A4	S100 calcium binding protein A4
SIBLING	Small integrin-binding ligand, N-linked glycoprotein
siRNA	Small interfering RNA
SNAIL1	Snail family transcriptional receptor 1
SNAIL2	Snail family transcriptional receptor 2
SPP1	Secreted phosphoprotein 1
SRA	Sequence read archive
TME	Tumor microenvironment
TMM	Trimmed mean of M-values
TP53	Tumor protein P53
TWIST	Twist family NHLH transcription factor
