# Supplementary material for: Investigating the Role of Osteopontin (OPN) in the Progression of Breast, Prostate, Renal and Skin Cancers
Source: Biomedicines. 2025 Jan 13;13(1):173. doi: 10.3390/biomedicines13010173 (PMC11762676; doi:10.3390/biomedicines13010173)
Supplement: Supplementary file 1 [file biomedicines-13-00173-s001.zip › biomedicines-3300281-supplementary.pdf]

Supplementary Information

Table S1. Samples analyzed for breast cancer.

Study	Cell types	Samples	Comparisons
GSE213474 [28]	MCF7 cells supplemented with heat-inactivated charcoal stripped FBS (HD); and HD with estradiol (E2)	30	Stimulation with IFN-γ for 6, 12, 24 and 48 hours v/s control
GSE186196 [29]	MCF7 and MDA-MB231 cells	18	Treatment with BKM and DMSO v/s control
GSE195816 [30]	MDA157 and MDA436 cells	12	Treatment with EBF1 shRNA v/s control
GSE190381 [31]	T47D cells containing 3 types of mutations (GATA splice-site deletion, C321 frame-shift, A333 frame-shift)	12	Mutated v/s control
GSE181460 [32]	T47D cells	6	LATS1/2 knockout v/s control

Table S2. Samples analyzed for prostate cancer.

Study	Cell types	Samples	Comparisons
GSE162294 [33]	LNCap (prostate adenocarcinoma) cells	36	Treatment with enzalutamide for 1, 3, 5, 7 and 14 days v/s control<br>Treatment with shTP53 (oligo #1 and #2) v/s control<br>Treatment with shBRCA1 (oligo #1 and #2) v/s control
GSE184676 [34]	LN95 (CPRC) cells	28	Treatment with R1881, exon7-targeted siRNA and AR-V7 exon-targeted siRNA v/s control
GSE133626 [35]	Prostate cancer tissue	16	Tumorous v/s control
GSE193127 [36]	VCaP and PC-3 cells	12	Transfected with FOXA1-targeting siRNA v/s non-targeting siRNA
GSE179990 [37]	PC-3 cells	9	Expressing NF-YA (short and long isoforms) v/s control

Table S3. Samples analyzed for renal cancer.

Study	Cell types	Samples	Comparisons
GSE167573 [38]	Renal cell carcinoma (RCC) tissue	28	Tumorous v/s control
GSE141295 [39]	Renal cell carcinoma (RCC) tissue	24	Affected by IgA nephropathy v/s control
GSE151419 [40]	Renal cell carcinoma (RCC) tissue	24	Tumor grade 2 v/s grade 3 and grade 4
GSE222245 [41]	786-O cells (wild-type and PBRM1 knockout)	20	Treatment with bortezomib (BTZ) for 9 hours v/s DMSO control<br>Treatment with SMARC2/4 ATPase inhibitor (BRM014) for 24 hours v/s DMSO control

Table S4. Samples analyzed for skin cancer.

Study	Cell types	Samples	Comparisons
GSE113113 [42, 43]	Cutaneous T-cell lymphoma (cuTCL) tissue	47	Tumor stage IA v/s IB, II and IVA2
GSE124857 [44]	MKL-1, MKL-2, MS-1, WaGa, PeTa, BroLi and UISO cells	42	Treatment with GSK-LSD1 inhibitor for 3 days, or CPI-670242 antibody for 1 day v/s control
GSE84293 [45]	Cutaneous squamous cell carcinoma (cuSCC) tissue	26	Non-lesional (NS) v/s actinic keratosis (AK) and cutaneous squamous cell carcinoma (SCC)
